# Distribution Pattern of Endangered *Cycas taiwaniana* Carruth. in China Under Climate-Change Scenarios Using the MaxEnt Model

**DOI:** 10.3390/plants14111600

**Published:** 2025-05-24

**Authors:** Chunping Xie, Meng Li, C. Y. Jim, Ruonan Chen

**Affiliations:** 1 Tropical Biodiversity and Bioresource Utilization Laboratory, Qiongtai Normal University, Haikou 571127, China; xcp@mail.qtnu.edu.cn (C.X.); 18184385406@163.com (R.C.); 2 Department of Social Sciences and Policy Studies, Education University of Hong Kong, Tai Po, Hong Kong, China; 3 Co-Innovation Center for the Sustainable Forestry in Southern China, College of Life Sciences, Nanjing Forestry University, Nanjing 210037, China; limeng@njfu.edu.cn

**Keywords:** *Cycas taiwaniana*, climate change scenario, MaxEnt model, bioclimatic variable, suitable habitat, conservation measure

## Abstract

Understanding the potential distribution patterns and habitat suitability of threatened species under climate change scenarios is essential for conservation efforts. This study aimed to assess the current and future distribution patterns of the endangered *Cycas taiwaniana* in China using the MaxEnt model under two contrasting climate change scenarios: SSP1-2.6 (low emissions) and SSP3-7.0 (high emissions), projected for the 2050s and 2070s periods. The model identified key bioclimatic variables influencing habitat suitability, including Annual Mean Temperature, Mean Diurnal Range, and Temperature Seasonality. Under current climate conditions, the species’ most suitable habitats are primarily located in southern coastal regions, with Hainan Island showing exceptional suitability. However, future projections under the moderate emission (SSP1-2.6) scenario suggest a significant shrinking of suitable habitat areas, particularly a 27.5% decline in excellent and a 35% decrease in good categories by the 2070s. In contrast, under the high-emission scenario (SSP3-7.0), while an initial decline in suitable habitats is projected, the model predicts an unexpected expansion of highly suitable areas by 2070, particularly in Guangxi, Guangdong, and Fujian coastal regions. The results highlight the vulnerability of *C. taiwaniana* to climate change and underscore the importance of developing adaptive conservation strategies to mitigate potential habitat loss. The findings also emphasize the need for further research on species-specific responses to climate change and the development of proactive measures to safeguard the future distribution of this threatened species.

## 1. Introduction

The geographic distribution of species is profoundly influenced by environmental factors, particularly climatic conditions, which play a crucial role in shaping the patterns over time [1,2]. At large scales, climatic factors such as temperature and precipitation serve as significant limiting factors for species ranges, creating spatial gradients imposing barriers to dispersal and curtailing the ability of species to colonize new areas [3,4,5]. At smaller scales, factors such as soil properties, topography, and biotic interactions, including competition and facilitation, further influence the fine-scale distribution patterns of plant species [6,7]. Biogeography research has long investigated biotic distribution patterns, providing insights into the mechanisms underlying species formation, evolution, and environmental adaptation [8,9]. These studies also enhance our understanding of biodiversity by elucidating the spatial distribution, ecological requirements, and vulnerability of species [10]. Global warming, coupled with human population growth and activities such as land conversion and resource extraction, has accelerated habitat reduction and species loss [11,12], presenting significant challenges to biodiversity conservation. Therefore, understanding the combined influences of climate change and human activities on species distribution is vital for developing effective conservation strategies [13,14].

Research indicates that climatic factors increasingly influence plant distributions at higher taxonomic levels and larger spatial scales [1,8]. Temperature seasonality and annual precipitation are identified as critical determinants of plant diversity patterns across different regions [15]. Evidence from fossil records and contemporary observations supports species shifting their ranges in response to climate change [16]. Temperature rise induces moving toward higher altitudes or higher latitudes [12,17]. Such conceptual and empirical studies underscore the importance of understanding climatic influences on habitat suitability and population dynamics [18,19]. The intricate relationship between environmental factors and species distribution highlights the need for more biogeography research. As global climate change continues to reshape ecosystems [20], comprehending these dynamics will be crucial for effective biodiversity conservation efforts [21]. Integrating climatic-influence knowledge with conservation strategies can better address the challenges posed by habitat loss and species extinction in the context of global environmental changes.

Species Distribution Models (SDMs) are statistical tools that integrate species occurrence data with environmental variables [14], such as climate, soil, and topography, to predict the potential geographic distribution of species in unexplored areas or under future climate scenarios [13,22]. SDMs are classified according to underlying algorithms and purposes [23]. They include association models (e.g., the Maximum Entropy model, MaxEnt, and the bioclimatic model, BIOCLIM) [22,24], mechanism models (e.g., CLIMEX and GARP) [25], hybrid models (e.g., Ecological Niche Factor Analysis, ENFA, and Bayesian Networks, BN) [26], and machine learning models (e.g., Random Forest, RF, and Support Vector Machines, SVM) [27].

Among the SDMs, MaxEnt has become one of the most widely used tools [17,28]. Its popularity stems from the ability to maintain accurate prediction despite small sample size, ease of use, flexibility in handling environmental variables, and user-friendly interface [22,29]. MaxEnt has demonstrated significant value in diverse domains such as species distribution prediction, ecological conservation, invasive species management, and climate change research [14]. With continual technological advancement, MaxEnt is expected to remain a pertinent tool in ecological studies and biodiversity conservation [13,24].

Cycads, among the most ancient gymnosperms and living seed plants, thrived from the late Triassic to early Cretaceous of the Mesozoic era before gradually declining by the late Cretaceous [30]. *Cycas* species are experiencing significant decline and have become one of the most threatened plant groups globally [31,32]. Research has shown that cycads are critical in creating habitable environments for adjunct organisms through their symbiotic relationship with soil microorganisms, particularly nitrogen-fixing cyanobacteria [33,34]. This symbiosis enhances nitrogen and carbon concentrations in the soil, fostering living conditions for other species. Losing cycads in a given ecosystem could usher serious consequences for biological communities that depend on their ecological services. The decline in soil chemistry and nutrient status can suppress the growth and development of other organisms [35]. Furthermore, cycads contribute to ecosystem diversity and resilience by creating unique microhabitats to raise spatial heterogeneity [36]. These intertwining inter-specific associations underscore cycads’ critical role in maintaining ecological balance and supporting biodiversity.

As an endangered species endemic to China, *Cycas taiwaniana* Carruth. (Cycadaceae family) is primarily distributed in subtropical and tropical South China provinces of Fujian, Guangdong, and Hainan [37]. Despite its relatively large extent of occurrence, covering 99,000 km^2^ [38], its habitat is severely threatened by habitat destruction because of urbanization, agricultural expansion, and environmental changes, leading to a significant decline in population and distribution range [39]. Field observations reveal that *C. taiwaniana* is highly adaptable to various soil types, suggesting that substrate is less limiting than climatic factors [40]. Wild plants are very rare, and the population is extremely small. Harvesting wild plants for ornamental use and trading also contributes to its decline [41]. Due to its ecological, cultural, and genetic significance, *C. taiwaniana* has become a focal point in current botanical and conservation biology research. Through molecular data [42], transcriptome analysis [43], and genetic structure studies [30], researchers have gradually clarified the species boundaries within the *C. taiwaniana* complex [44], uncovering the historical background of genetic differentiation and the influence of climate and geographical factors.

Investigating the population structure of *C. taiwaniana* across various habitats has identified human disturbance as the most critical factor affecting its survival [18]. The International Union for Conservation of Nature (IUCN) Red List of Threatened Species included *C. taiwaniana* as an Endangered species in 2020, marked by a decreasing population and continuing decline of mature individuals [38]. China has designated it a Class I protected wild plant [39]. The fragmentary surviving subpopulations are distributed in remote and largely inaccessible sites, including some protected areas. Understanding the geographical distribution pattern of *C. taiwaniana* and the impact of climate change is essential for identifying key habitats, assessing climate-induced risks and range changes, and establishing a scientific foundation for effective conservation strategies.

This study examines the distribution patterns of *C. taiwaniana* in China using the MaxEnt model under both current climate and future climate change scenarios. It addresses a critical research gap, specifically the lack of predictive distribution models for *C. taiwaniana* under climate-change scenarios. The specific objectives are: (1) to identify and assess the key climatic factors influencing its current distribution; (2) to predict potential habitats for the species’ future growth based on current distribution data; (3) to project changes in its suitable habitats under climate change scenarios. The results aim to support the conservation of the invaluable species, promote its sustainable management, and provide an objective basis to select appropriate sites for ex situ conservation.

## 2. Results

### 2.1. Model Accuracy

**Figure 1:**
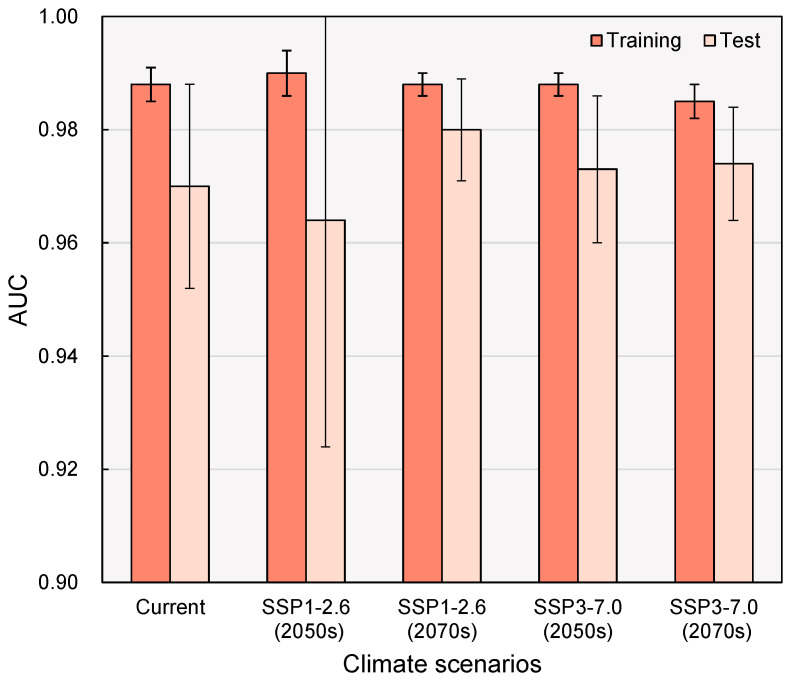
AUC values of habitat distribution from two different scenarios (SSP1-2.6 and SSP3-7.0) in current and two future periods (2050s and 2070s). The error bar represents the standard deviation.

The AUC values for the habitat distribution of *C. taiwaniana* across different climate change scenarios and periods indicate a generally high model performance (AUC > 0.9) (Figure 1), suggesting reliable predictions of species distribution. In the current climate, the training AUC value is 0.988 ± 0.003, and the test AUC is 0.970 ± 0.018. They both express excellent model performance. Under future climate scenarios, the model performance remains strong. For the 2050s under the SSP1-2.6 scenario, the training AUC is 0.990 ± 0.004, and the test AUC is 0.964 ± 0.040, while under the SSP3-7.0 scenario, the training AUC is 0.988 ± 0.002, with a test AUC of 0.980 ± 0.009. For the 2070s, the model performance slightly declines, with AUC values of 0.985 ± 0.003 for training and 0.974 ± 0.010 for the test set under the SSP3-7.0 scenario, and 0.988 ± 0.002 and 0.973 ± 0.013 for training and test AUC, respectively, under the SSP3-7.0 scenario. These high AUC values across all scenarios and periods indicate that the MaxEnt model provides consistent and reliable predictions of *C. taiwaniana* habitat distribution under current and future climate change conditions.

### 2.2. Main Climatic Factors Shaping Distribution Pattern

**Figure A1:**
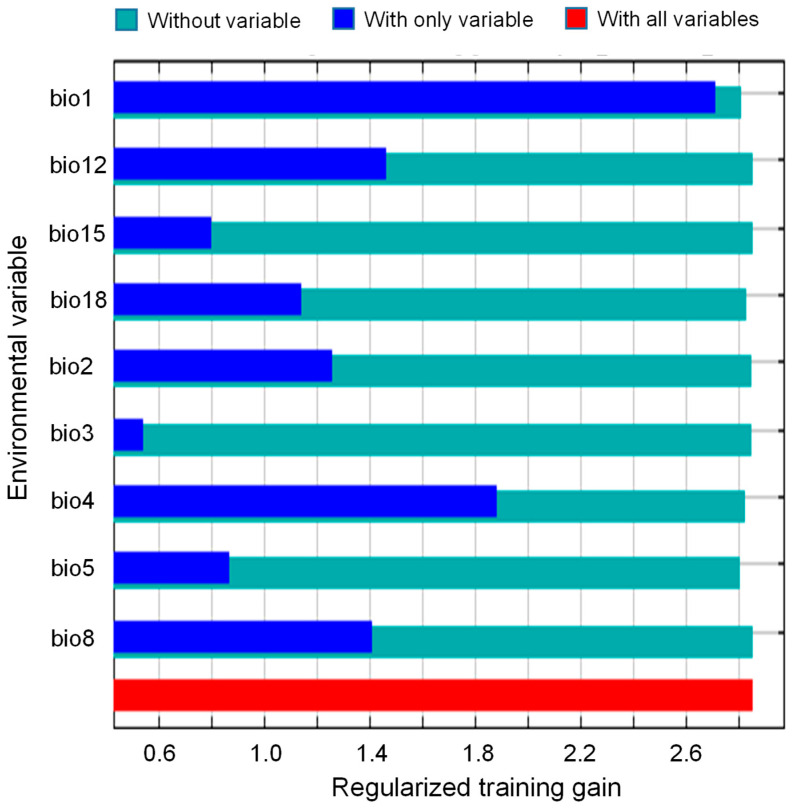
Results of the jackknife test assessing variable importance in the *Cycas taiwaniana* habitat distribution model. The environmental variable bio1 (annual mean temperature) yields the highest gain when used alone, indicating it provides the most valuable information independently. Values represent averages across replicate runs.

The distribution of *C. taiwaniana* in China is shaped by a range of bioclimatic factors, with temperature-related variables showing more consistency across its habitat (Table 1). The relatively low coefficients of variation in parameters such as the annual mean temperature (bio1, 11.62%) and max temperature of the warmest month (bio5, 6.55%) suggest that these temperature parameters remain relatively stable across the species distribution range [5]. These stable temperature variables play a dominant role in defining suitable habitats for *C. taiwaniana*. In contrast, precipitation-related variables have higher coefficients of variation, particularly the annual precipitation (bio12, 28.31%) and precipitation of the warmest quarter (bio18, 26.99%), indicating considerable variations in rainfall patterns across the species range. While precipitation variability is important, temperature parameters have a more consistent and stronger influence in determining the species distribution. The jackknife test results (Figure A1) reveal that bio1, bio4, bio12, bio8, and bio2 exhibit the highest gain when used independently, indicating these bioclimatic variables provide unique information not captured by other variables. The impact of temperature stability is likely more critical in shaping the habitats of *C. taiwaniana*, while precipitation may have a more supplementary, context-dependent role.

**Figure 2:**
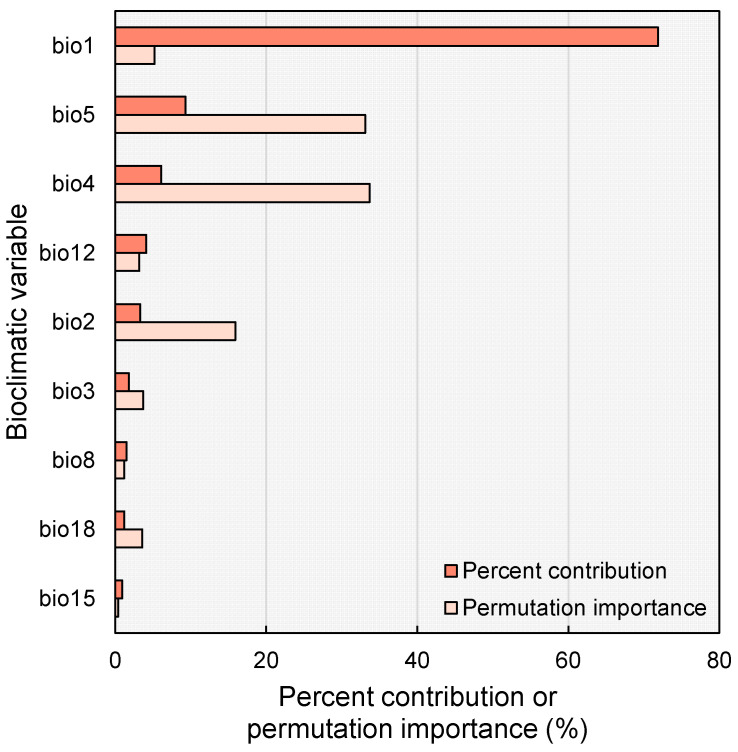
Percent contributions and permutation importance of the nine bioclimatic variables included in the MaxEnt model. Refer to Table 1 for the meaning of the bioclimatic variables.

The MaxEnt modeling results for *C. taiwaniana* reveal the relative importance of nine bioclimatic variables in predicting the species distribution (Figure 2). Temperature-related variables, particularly bio1, stand out with the highest percent contribution of 71.9%, indicating their dominant role in shaping the species’ habitat suitability. In contrast, precipitation and seasonality variables, such as bio5 and bio4 (temperature seasonality), show significant permutation importance of 33.1% and 33.7%, respectively, suggesting their considerable influence on the model’s predictive accuracy. While bio12 and bio2 (mean diurnal range) contribute to the model, their percent contributions (4.1% and 3.3%) are relatively lower, reflecting their secondary role in defining suitable habitats for *C. taiwaniana*. Variables such as bio3 (isothermality), bio8 (mean temperature of wettest quarter), bio18, and bio15 (precipitation seasonality) contribute even less, indicating that they have a minor effect on the species distribution under the climate-change scenarios modeled. These findings underscore the critical role of temperature variables in determining the distribution of *C. taiwaniana*, with precipitation and seasonality factors playing a supplementary yet important role in habitat prediction.

**Figure 3:**
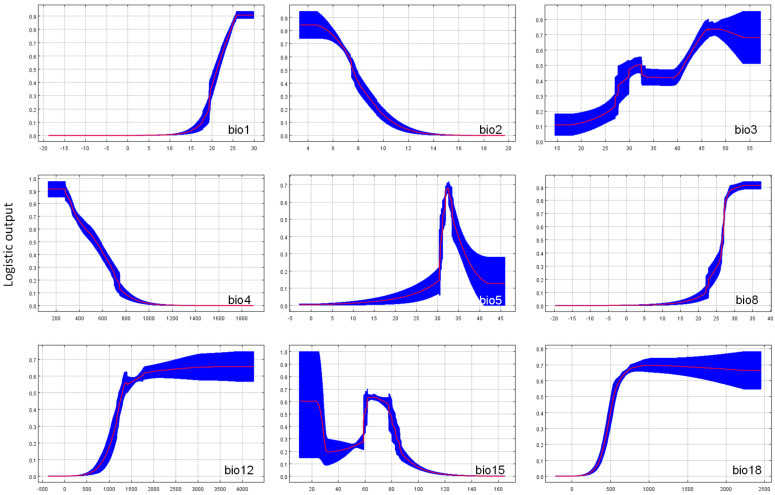
Response curves of nine bioclimatic predictor variables used in the MaxEnt model for *Cycas taiwaniana*. The curves show the mean response of the 10 replicate MaxEnt runs (red line) and the mean +/− one standard deviation (blue belt). Refer to Table 1 for the meaning of the bioclimatic variables.

Several crucial bioclimatic variables exhibit distinct patterns in determining species habitat suitability based on the MaxEnt response curves (Figure 3). Variable bio1 demonstrates a pronounced sigmoid response curve with a definitive threshold near 20 °C, close to the actual current distribution area, where habitat suitability increases dramatically between 15 and 25 °C, with minimal suitability below 15 °C and optimal conditions above 25 °C, as indicated by the narrow blue margin suggesting high prediction reliability. Variable bio2 displays a declining response curve, indicating optimal conditions at lower diurnal temperature ranges (4–8 °C), with reduced species tolerance for areas experiencing high daily temperature fluctuations. However, the broader blue margin at higher values suggests some uncertainty. Notably, bio4 shows a clear negative relationship with habitat suitability, peaking at low seasonality values (0–400 units) before sharply declining, with minimal occurrence probability in highly seasonal environments exceeding 1000 units. Collectively, these patterns suggest the species is well-adapted to environments characterized by warm, stable annual temperatures (approximately 25 °C), minimal daily temperature fluctuations, and low seasonal temperature variability, indicating a likely adaptation to tropical or subtropical environments with rather consistent temperature conditions throughout the year.

### 2.3. Current Species Range Distribution Pattern

**Figure 4:**
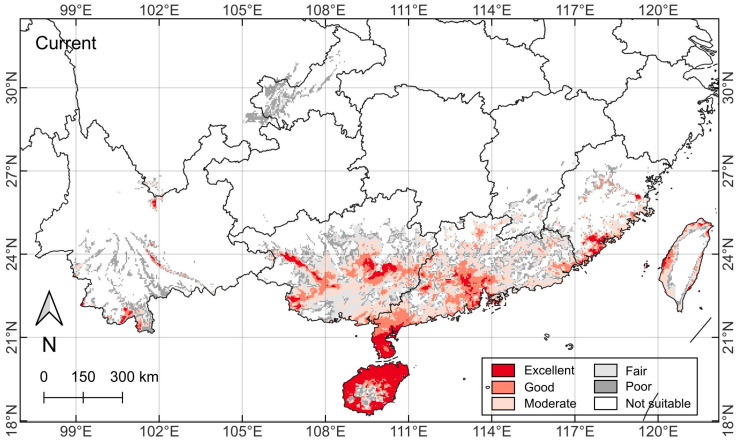
Potential suitability areas of *Cycas taiwaniana* under the current climate scenario in China (1970–2000) divided into six categories based on the calculated habitat suitability index. Refer to Figure 6 for the locations and names of the provinces.

*C. taiwaniana* exhibits a geographically restricted distribution pattern in southern China, primarily occurring between 18° N–27° N latitude and 105° E–122° E longitude (Figure 4). Supported by field observations, the species is predominantly distributed across six southernmost provinces. Three provinces of Guangxi, Guangdong, and Hainan constitute the core distribution areas, with the bulk of their lands designated as extensive and rather continuous potential suitability areas. In Hainan, the distribution fills the island continuously, except for the scattered patches in the central high mountains. In Guangxi and Guangdong, the southern portion of the distribution has a more continuous pattern, which becomes progressively less continuous toward the northern portion.

Yunnan, Fujian, and Taiwan contribute to the marginal species range, with discontinuous suitable habitats covering only a small proportion of their lands. In Yunnan, the distribution is mainly in its southern portion with lower elevation, sheltered in long valleys intercalated between elongated mountain ranges. In Fujian, more distribution with a more continuous spread is found along the coastal belt than inland areas with more scattered and fragmented patches. In Taiwan, the distribution is restricted to the coastal belt wrapping around the island and excluded from the central mountains.

Hainan, the Leizhou Peninsula of Guangdong (north of Hainan), and a portion of south Yunnan fall under the peripheral and middle tropical zones [45]. This is the major land area of China with a tropical climate. The remaining parts of the study area are subsumed under the southern subtropical zone [45]. The regional monsoon climate influences the main distribution area with hot summers and warm winters, abundant rainfall, and relatively stable seasonal conditions. The climatic profile aligns well with the species’ ecological preferences for warm, humid environments with moderate temperature fluctuations.

### 2.4. Current Habitat Suitability Pattern

The habitat suitability modeling under current climate conditions (1970–2000) reveals a highly differentiated potential distribution pattern for *C. taiwaniana* (Figure 4). The excellent suitable areas, covering 4.0 × 10^4^ km^2^, are predominantly concentrated in areas south of latitude 21° N. They cover rather contiguously a large proportion of Hainan Island except for its interior high mountains and the Leizhou Peninsula to its north. These two core excellent areas represent the largest stretch of the most tropical parts of China. Away from the core distribution, the excellent habitats are small and more fragmented patches along the southeastern coastal and some inland areas, where local climatic conditions optimally meet the species’ requirements (Table 2). Guangdong and Guangxi accommodate the bulk of the excellent habitats. Fujian and Taiwan have scattered patches, and Yunnan has a few small pockets.

The good (6.0 × 10^4^ km^2^) and moderate (8.5 × 10^4^ km^2^) suitability zones are notably larger than the excellent ones. They extend mainly through Guangxi and Guangdong, accompanied by small and geographically confined patches in Fujian, Taiwan, and Yunnan. They tend to lie contiguous to the excellent habitats, sometimes wrapping around them. In Fujian and Taiwan, the small amount of good and moderate habitats are restricted to the coastal belt.

The fair (8.2 × 10^4^ km^2^) and poor (9.3 × 10^4^ km^2^) habitats are distributed mainly in the peripheral areas of the six southern provinces. Compared with excellent, good, and moderate habitats, their distribution pattern is characterized by many small and fragmented patches. In the core provinces of Guangxi, Guangdong, and Hainan, they become progressively more common away from the coast and toward the northern part. In Fujian, they are detached from the more favorable habitats along the coastal belt. In Taiwan, they form a narrow strip bordering the better habitats. In Yunnan, they are the dominant habitat types that follow the narrow valley configuration. Outside the six southern provinces, the outlier Chongqing is the only place with a noteworthy distribution in the principally fair to poor categories, indicating the marginal growth conditions. Sichuan and Jiangxi have only small and fragmented pockets. Notably, most of the study area (925.6 × 10^4^ km^2^) is classified as unsuitable, particularly in the northern and inland mountainous regions.

The distribution of habitat classes is consistent with the species’ observed response curves to key bioclimatic variables (Figure 3). This distribution pattern strongly suggests that *C. taiwaniana* is a climate-sensitive species with specific environmental requirements, primarily thriving in subtropical and tropical areas with stable, warm temperatures and adequate precipitation levels typical of China’s southern coastal regions.

### 2.5. Future Potential Distribution

**Figure 5:**
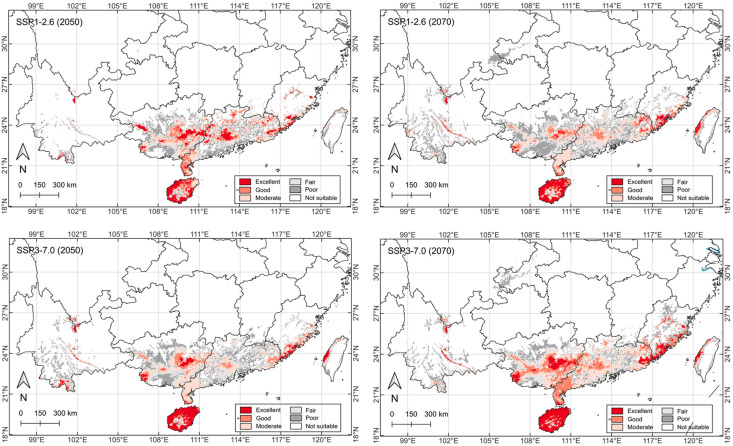
MaxEnt modeling of potential suitability areas for *Cycas taiwaniana* based on two future climate-change scenarios in the 2050s and 2070s (columns) at SSP1-2.6 and SSP3-7.0 (rows). Habitat suitability is divided into six categories based on the calculated habitat suitability index. Refer to Figure 6 for the locations and names of the provinces.

Under future climate change scenarios, the habitat suitability distribution of *C. taiwaniana* shows notable spatial and temporal variations across different regions of China. The analysis reveals distinct differences between SSP1-2.6 and SSP3-7.0 scenarios across the 2050s and 2070s periods, with significant changes in the distribution and quality of suitable habitats compared with current conditions (Figure 4 and Figure 5).

The quantitative assessment indicates a general declining temporal trend in high-quality habitats for the SSP1-2.6 scenario (Table 2 and Figure 5). Under SSP1-2.6, excellent suitable areas are projected to decrease from the current 4.0 × 10^4^ km^2^ to 3.5 in the 2050s and 2.9 in the 2070s. However, SSP3-7.0 shows a contrasting pattern, increasing to 5.0 × 10^4^ km^2^ in the 2070s. Hainan remains the most amenable province by maintaining most of its largely contiguous excellent habitats despite climatic changes brought by the two scenarios in both periods. Similarly, good suitable areas show substantial fluctuations, declining significantly under SSP1-2.6 in both periods but increasing under SSP3-7.0 in the 2070s. The overall average changes indicate shrinkage for favorable categories of excellent (−8.5%), good (−18.4%), and moderate (−11.0%), while fair and poor suitability areas show expansions of 5.2% and 10.8%, respectively. In general, the 2050s will experience more habitat shrinkage than the 2070s, and the SSP1-2.6 will bring more habitat loss than SSP3-7.0.

Spatially, the most pronounced changes are observed in the coastal regions of southern China. Hainan Island maintains relatively stable excellent suitability across scenarios and years, while the southeastern coastal areas show varying shifts in habitat quality (Figure 5). The distribution patterns suggest a potential northward move of suitable habitats under SSP3-7.0 by the 2070s. This is particularly evident in the increased excellent and good suitability areas in parts of Guangdong and Fujian provinces. Meanwhile, the western populations in Yunnan show relatively consistent but limited suitable areas across different scenarios, indicating these regions might serve as potential climate refugia for the species.

## 3. Discussion

### 3.1. Current Potential Suitability Habitats and Key Bioclimatic Variables

The potential distribution of *C. taiwaniana* under current climate conditions is shaped by the key bioclimatic variables listed in Table 1, with temperature-related factors playing a particularly crucial role in determining habitat suitability (Figure 2 and Figure A1). Analysis of the MaxEnt model response curves (Figure 3) reveals that bio1 (annual mean temperature) denotes a distinct sigmoid relationship with habitat suitability, showing a sharp increase in the probability of occurrence between 15 and 25 °C (Figure 3). This is largely consistent with the distribution patterns of *Cycas* species in China, suggesting that south subtropical-tropical regions serve as the center of diversity for *Cycas* in the country [37,39]. This strong temperature dependence aligns with the species’ observed distribution pattern along China’s southernmost regions, where warm subtropical to tropical conditions prevail [46].

Diurnal and seasonal temperature variations also emerge as critical factors influencing the species distribution [47]. Factor bio2 (mean diurnal range)demonstrates an inverse relationship with habitat suitability [48], indicating the species’ preference for environments with limited daily temperature fluctuations. Similarly, bio4 (temperature seasonality) shows a clear negative correlation with habitat suitability, with optimal conditions occurring in areas of low seasonal variability (Figure 3). Similar patterns have also been observed in *Cycas sexseminifera* [49] and *Ceratozamia* spp. [50]. These patterns suggest that *C. taiwaniana* is particularly sensitive to temperature stability, thriving in regions with minimal daily and seasonal temperature fluctuations.

The interaction of these bioclimatic variables creates a complex environmental envelope that effectively restricts the species’ potential distribution to specific geographic regions [5,24,26]. The most suitable habitats are concentrated in areas where these key variables converge within optimal ranges [2,8], particularly in coastal regions of southern China, including Hainan Island, southeastern Guangdong, and parts of Fujian province (Figure 1 and Figure 4). The strong influence of temperature-related variables explains why the species’ potential distribution is largely confined to latitudes below 30° N, where stable, warm temperatures predominate throughout the year [51]. The species’ sensitivity to temperature variables suggests it may be particularly vulnerable to climate change [31,49], especially in areas where projected changes could push conditions beyond the species’ tolerance thresholds.

### 3.2. Spatial Distribution Under Future Climate Change Scenarios

The spatial distribution patterns of *C. taiwaniana* under future climate scenarios reveal significant temporal and spatial shifts (Figure 5), with varying responses under different Shared Socioeconomic Pathways (SSPs). Under the moderate emission scenario (SSP1-2.6), suitable habitats show a consistent declining trend, with excellent and good suitability areas decreasing by 27.5% and 35%, respectively, by the 2070s compared with current conditions (Table 2). However, under the high emission scenario (SSP3-7.0), a more complex pattern emerges, with an initial decline followed by an expansion of highly suitable areas by the 2070s, particularly in the coastal regions of Guangdong and Fujian provinces.

These distribution shifts can be primarily attributed to the species’ specific temperature requirements and their interaction with projected climate changes. The observed expansion of suitable habitats under SSP3-7.0 by 2070s aligns with previous studies on tropical and subtropical species’ responses to warming scenarios [52,53]. This pattern’s underlying mechanism likely involves the northward shift of thermal isolines [54], creating new potentially suitable habitats in previously marginal areas. However, this apparent advantage may be offset by increased climate variability and extreme weather events [55], which could suppress the species’ establishment and survival despite seemingly suitable average conditions [56].

The contrasting responses under different scenarios highlight the complex interplay between climate change and species distribution dynamics [57]. The reduction in suitable habitats under SSP1-2.6 suggests that even moderate climate change could disrupt the delicate balance of conditions required by *C. taiwaniana*, particularly regarding temperature stability and seasonal patterns [58]. This finding is consistent with studies on other cycad species that demonstrate high sensitivity to climate fluctuations [32]. The projected increase in poor and fair suitability areas at the expense of excellent, good, and moderate areas across all scenarios indicates a general trend toward habitat degradation, which could significantly affect the species’ long-term survival (Table 2). Many endangered plants are highly sensitive to environmental changes [59], and their evolutionary adaptations often cannot keep pace with the rapid shifts in critical climatic parameters [60]. As a result, climate change tends to cause more harm than good for these species [17], exacerbating their vulnerability and threatening their survival in changing conditions. Furthermore, the fragmentation of suitable habitats observed in both scenarios could create isolated populations [61], potentially reducing genetic exchange and increasing vulnerability to local extinction events [62]. These undesirable changes underscore the importance of considering both direct climate effects and indirect ecological consequences when planning conservation strategies for this species under future climate scenarios.

### 3.3. Model Assessment

**Figure 6:**
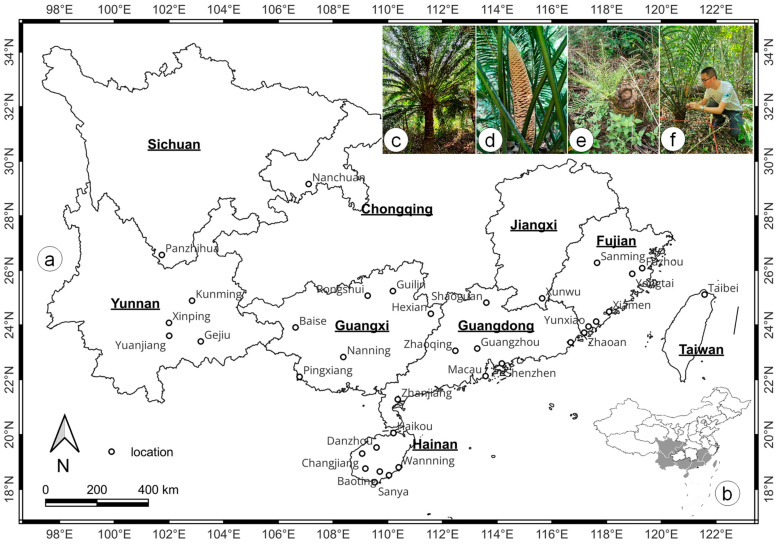
Distribution and habitats of *Cycas taiwaniana* in China. (**a**) Natural distribution across nine provinces and municipalities, (**b**) overview of distribution within China, (**c**) wild growth habitat, (**d**) male cone, (**e**) damaged individual, and (**f**) field investigation site.

In species distribution modeling, sample size critically influences prediction accuracy and reliability, particularly when using presence-only models such as MaxEnt [24,63,64]. The study of *C. taiwaniana*, with a sample size of only 36 occurrence records (Figure 6), exemplifies a situation where MaxEnt’s predictive power remains robust despite limited data. Although many modeling techniques struggle with small sample sizes, resulting in high variability and lower accuracy, MaxEnt has performed well even with a small sample of occurrence data [65]. Several studies have highlighted MaxEnt’s ability to generate reliable predictions with small datasets [63,66], especially compared with other algorithms such as Bioclim, Domain, or GARP, which suffer from reduced predictive accuracy as sample sizes decrease [65,66].

Research has demonstrated that MaxEnt maintains strong predictive performance even with sample sizes as low as 10 to 25 presence points [67,68]. This is particularly relevant when studying species with limited geographic distribution, where occurrences may be sparse. MaxEnt’s effectiveness in these situations is partly due to its ability to model environmental relationships based on presence-only data [14,22] without requiring absence data. This feature, coupled with the algorithm’s flexible handling of complex interactions between environmental variables [23], allows MaxEnt to make reliable predictions even when data are scarce [17]. In the case of *C. taiwaniana*, despite the small sample size, MaxEnt provided high-quality predictions of suitable habitats across China under current and future climate scenarios (Figure 1). The AUC values in this study were consistently high, indicating good model performance and a low omission rate. However, it is important to note that while small sample sizes do not necessarily compromise the model’s ability to predict species distribution [28], the reliability of these predictions increases when the modeling process incorporates a careful selection of environmental variables and appropriate parameter tuning [69].

### 3.4. Implication for Conservation

The projected changes in habitat suitability for *C. taiwaniana* under future climate scenarios necessitate a comprehensive, multi-faceted conservation strategy to ensure the species’ long-term survival. Priority should be given to protecting and managing existing populations in areas that maintain high habitat suitability across different climate scenarios [70], particularly in Hainan Island and coastal regions of Guangxi, Guangdong, and Fujian provinces [18], which could serve as critical climate refugia [43,44]. These areas should be designated as protected zones with enhanced management protocols to minimize anthropogenic disturbances and maintain habitat quality [71].

Ex situ conservation measures should be strategically implemented to complement in situ protection [37,39]. Establishing germplasm banks and botanical gardens in climatically suitable areas can help preserve genetic diversity and provide source material for future restoration efforts [72,73]. Additionally, assisted migration programs should be considered for populations in areas projected to become unsuitable [50], with careful selection of recipient sites based on both current and projected future climate suitability [70]. This approach requires thorough genetic and ecological assessments to ensure population viability and minimize potential negative impacts on recipient ecosystems.

To enhance the adaptive capacity of *C. taiwaniana* populations, conservation strategies should focus on maintaining, restoring, and creating habitat connectivity [61], particularly along coastal areas where suitable conditions are projected to persist. This could involve the creation of ecological corridors and stepping-stone habitats to facilitate gene flow between fragmented populations [62], thereby increasing genetic diversity and adaptive potential [44]. Furthermore, local-scale habitat management practices should be implemented to buffer against climate extremes [71], such as maintaining or enhancing canopy cover to moderate temperature fluctuations and establishing microhabitat features that provide climate refuge at small spatial scales [74].

The success of these conservation measures heavily depends on integrated research and monitoring programs. Long-term demographic studies, genetic monitoring, and regular population viability assessments under changing climatic conditions are essential [75]. Collaboration between research institutions, conservation organizations, and local communities is crucial for the effective implementation of conservation strategies [76]. Public education and stakeholder engagement programs should be developed to raise awareness about the ecological and cultural significance of *C. taiwaniana* and promote community-based conservation initiatives [71]. Such comprehensive approaches will be vital for ensuring the survival of this species in the face of climate change and human perturbation while maintaining its ecological and evolutionary potential.

### 3.5. Limitations of the Study

This study is limited by its lack of analysis on the spatial overlap between predicted “excellent” habitat suitability areas for *C. taiwaniana* and regions with intense human activities, such as urban or rural landscapes, which could significantly curtail conservation feasibility. Urbanization, agricultural expansion, and other anthropogenic pressures may degrade habitat quality or limit access to suitable areas, potentially rendering some predicted habitats unsuitable for long-term conservation. Species distribution models that rely solely on climatic variables often overestimate habitat suitability by overlooking non-climatic factors, such as land-use changes and human-induced disturbances [77]. Habitat fragmentation driven by human activities, including urban development and ornamental harvesting, has significantly reduced cycad populations in regions such as Hainan and Guangdong [30]. The distribution of *C. taiwaniana* in Fujian, Guangdong, and Hainan often coincides with areas experiencing increasing land-use pressure, highlighting the need to incorporate anthropogenic factors into conservation planning [18,39]. Although the MaxEnt model employed here provides a robust climatic framework for identifying potential habitats under current and future climate scenarios, it does not account for the dynamic impacts of human activities, which could compromise the practical suitability of predicted areas [78]. For example, highly suitable habitats near urban centers may be threatened by infrastructure development, while rural areas may face challenges from agricultural intensification. This limitation suggests that viable conservation site availability may be overestimated without integrating spatial analysis of land-use patterns [79]. Future research should combine high-resolution land-use and urbanization data with climatic variables to refine habitat suitability assessments and prioritize conservation efforts in areas with minimal anthropogenic disturbance. Such an integrated natural-cum-anthropogenic approach would enhance the applicability of these findings for developing targeted in situ and ex situ conservation strategies for *C. taiwaniana*, ensuring alignment with real-world ecological and socioeconomic constraints.

## 4. Materials and Methods

### 4.1. Collecting Current C. taiwaniana Distribution Data

We collected and compiled 872 distribution records of *C. taiwaniana*, covering its current geographic range. The data sources included: (1) seven field surveys conducted by the research team, primarily focusing on distribution locations in Hainan Island, including Ledong, Lingshui, Baoting, Wanning, Danzhou, Haikou, and Changjiang; (2) distribution recorded in the relevant literature accessed through databases such as China National Knowledge Infrastructure (CNKI); (3) specimen records retrieved from online databases, primarily the National Specimen information infrastructure (NSII, http://www.nsii.org.cn/2017/home.php, accessed on 17 September 2024), for ‘*Cycas taiwaniana*’ and its synonyms (e.g., *Cycas hainanensis* C. J. Chen, *Cycas ramiflora* G. A. Fu, *Cycas lingshuigensis* G. A. Fu, *Cycas changjiangensis* N. Liu, *Cycas pectinata* subsp. *manhaoensis* C. Chen & P. Yun, *Cycas revoluta* var. *taiwaniana* (Carruth.) J. Schust., *Cycas hainanensis* subsp. *changjiangensis* (N. Liu) N. Liu [80]). Duplicate records and entries with unclear location descriptions were excluded from this study. Additionally, only one record per county-level region was retained, resulting in 36 records used as the base for this study’s dataset (Figure 6). An alternative 5 km × 5 km grid-based approach was tested, yielding a comparable number of records, confirming the robustness of our sampling strategy. Latitude and longitude coordinates were obtained using Baidu’s coordinate-picking query tool (https://api.map.baidu.com/lbsapi/getpoint/index.html, accessed on 22 November 2024), with the coordinate system set to WGS 1984.

### 4.2. Selecting Climate Scenarios and Environmental Variables

Current and future climate data were sourced from WorldClim (http://www.worldclim.org, accessed on 7 June 2023) [81], with future climate projections selected from the Coupled Model Intercomparison Project Phase 6 (CMIP6) and the Beijing Climate Center Climate System Model (BCC-CSM2-MR) [82], both at a resolution of 2.5′. Future climate data from CMIP6 include five Shared Socioeconomic Pathway (SSP) scenarios: SSP1 (sustainability, taking the green road), SSP2 (middle of the road), SSP3 (regional rivalry, a rocky road), SSP4 (inequality, a road divided), and SSP5 (fossil-fueled development, taking the highway) [83]. This study focused on three scenarios: current, SSP1-2.6 representing the moderate emission scenario, and SSP3-7.0 representing the high emission scenario. Changes in the suitable habitats of *C. taiwaniana* were simulated in two periods, namely the 2050s and 2070s.

To eliminate multicollinearity among environmental variables, Pearson correlation analysis was performed using SPSSAU (https://spssau.com/index.html, accessed on 11 January 2025) for the 19 bioclimatic variables (bio1–bio19) (Table A1). When the correlation coefficient |*r*| ≥ 0.8 [64] (Table A2), we retained the variable with the greatest ecological relevance to *C. taiwaniana*, determined based on its known physiological and distributional responses to climate, as informed by prior studies in the *Cycas* genus [49,84]. Consequently, nine environmental variables were retained for modeling: bio1, bio2, bio3, bio4, bio5, bio8, bio12, bio15, and bio18.

### 4.3. Spatiotemporal Mapping of Suitable Habitats

To model the distribution of *C. taiwaniana*, MaxEnt (version 3.4.4) was implemented using a 10-fold cross-validation approach with 36 occurrence records [85]. In each run, 90% of the records were used for model training, and 10% were reserved for testing, repeated across 10 runs to ensure robust model performance. The parameter optimization method for the MaxEnt model was conducted following the approach outlined in the reference [86]. Bioclimatic variables were selected after eliminating multicollinearity via Pearson correlation analysis (|*r*| < 0.8), and model accuracy was assessed using the Area Under the Curve (AUC) metric, with values exceeding 0.9 indicating high predictive performance.

The ASCII files generated by the MaxEnt simulations were converted into Raster format, with raster data representing the survival probability of *C. taiwaniana*. Using the reclassification tool in the spatial analysis toolbox, habitat suitability was divided into six ordinal categories: not suitable (0), poor (0–0.15), fair (0.15–0.30), moderate (0.30–0.45), good (0.45–0.60), and excellent (>0.60), based on logistic suitability scores and prior studies of other species [17,87], to provide a nuanced delineation of potential suitable areas for *C. taiwaniana* conservation. Furthermore, we assessed the importance of variables in the final model through the Jackknife method [88].

## 5. Conclusions

This study provides useful insights into the potential impacts of climate change on the endangered *C. taiwaniana* distribution in China, revealing complex patterns of habitat suitability shifts under different climate scenarios. The findings demonstrate that *C. taiwaniana* is highly sensitive to temperature-related variables, making it particularly vulnerable to climate change impacts. The projected reduction in suitable habitats under moderate emission scenarios and potential habitat fragmentation and shrinkage pose significant challenges to the species’ long-term survival. However, identifying stable, suitable areas across scenarios, particularly in Hainan Island and specific coastal regions of other southern provinces, provides valuable information for conservation planning. Based on these findings, we recommend a multi-faceted conservation approach including: (1) prioritizing the protection of identified climate refugia, (2) implementing assisted migration programs for vulnerable populations, (3) establishing ex situ conservation facilities in climatically suitable areas, and (4) developing habitat connectivity corridors along coastal regions. Future conservation efforts should focus on integrating these strategies with long-term monitoring programs and community-based initiatives to ensure the species’ persistence under changing climatic conditions. This study’s methodology and findings can serve as a reference for similar research on other threatened species facing climate change challenges.

## Figures and Tables

**Table 1:** Descriptive statistical profile of the nine bioclimatic parameters in the distribution range of *C. taiwaniana* in China.

Bioclimatic Variable	Bioclimatic Attribute	Mean ± SD	Minimum	Maximum	95% Confidence Interval	Coefficient of Variation (%)
bio1	Annual mean temperature	21.7 ± 2.5	15.4	25.8	20.8–22.5	11.62
bio2	Mean diurnal range (Mean of monthly (max temp-min temp))	7.8 ± 1.7	5.1	12.4	7.3–8. 4	22.11
bio3	Isothermality (bio2/bio7) (×100)	36.1 ± 7.9	24.8	49.5	33.5–38.7	21.91
bio4	Temperature seasonality	528.5 ± 134.9	279.2	756.9	484.4–572.5	25.52
bio5	Max temperature of warmest month	31.8 ± 2.1	24.5	34.4	31.1–32.5	6.55
bio8	Mean temperature of wettest quarter	26.1 ± 2.3	20.2	29.3	25.3–26.8	9.01
bio12	Annual precipitation	1449.2 ± 410.2	862.0	3207.0	1315.2–1583.2	28.31
bio15	Precipitation seasonality	71.0 ± 14.3	19.2	99.2	66.4–75.7	20.12
bio18	Precipitation of warmest quarter	585.6 ± 158.1	391.0	1128.0	534.0–637.2	26.99

**Table 2:** Predicted suitable areas (× 10^4^ km^2^) for *Cycas taiwaniana* under the current and future climate scenarios are classified into six suitability categories generated by the MaxEnt model.

Climate Scenario	Current	SSP1-2.6 (2050s)	SSP1-2.6 (2070s)	SSP3-7.0 (2050s)	SSP3-7.0 (2070s)	Average ^a^	Change ^b^
Excellent	4.0	3.5	2.9	3.4	5.0	3.7	−8.5
Good	6.0	5.1	3.9	3.3	7.4	4.9	−18.4
Moderate	8.5	6.9	7.8	7.2	8.4	7.6	−11.0
Fair	8.2	7.3	10.1	9.0	8.1	8.6	5.2
Poor	9.3	7.6	13.3	10.6	9.9	10.4	10.8
Not suitable	925.6	931.4	923.8	928.2	922.9	926.6	0.1

^a^ The average value of suitable habitat areas projected under future climate change scenarios. ^b^ Comparison of suitable habitat areas under the current climate scenario and the average values projected under future climate change scenarios.

## Data Availability

Data in this study are available at https://doi.org/10.57760/sciencedb.19432.

## Appendix A

**Table A1:** Nineteen bioclimatic variables initially included to predict the distribution patterns of *Cycas taiwaniana*. The nine highlighted variables were selected by multicollinearity test for MaxEnt modeling.

Bioclimatic Variable	Unit	Bioclimatic Variable	Unit
**bio1: Annual mean temperature**	°C	bio11: Mean temperature of the coldest quarter	°C
**bio2: Mean diurnal range**	°C	**bio12: Annual precipitation**	mm
**bio3: Isothermality**	Index	bio13: Precipitation of the wettest month	mm
**bio4: Temperature seasonality**	Index	bio14: Precipitation of the driest month	mm
**bio5: Max temperature of the warmest month**	°C	**bio15: Precipitation seasonality**	Index
bio6: Min temperature of the coldest month	°C	bio16: Precipitation of the wettest quarter	mm
bio7: Temperature annual range	°C	bio17: Precipitation of the driest quarter	mm
**bio8: Mean temperature of the wettest quarter**	°C	**bio18: Precipitation of the warmest quarter**	mm
bio9: Mean temperature of the driest quarter	°C	bio19: Precipitation of the coldest quarter	mm
bio10: Mean temperature of the warmest quarter	°C		

**Table A2:** Pearson correlation coefficient matrix of the 19 bioclimatic variables (described in Table A1).

	bio1	bio2	bio3	bio4	bio5	bio6	bio7	bio8	bio9	bio10	bio11	bio12	bio13	bio14	bio15	bio16	bio17	bio18	bio19
bio1	1																		
bio2	−0.337 *	1																	
bio3	0.255	0.628 **	1																
bio4	−0.567 **	−0.043	−0.793 **	1															
bio5	0.545 **	−0.195	−0.337 *	0.330 *	1														
bio6	0.934 **	−0.513 **	0.244	−0.695 **	0.297	1													
bio7	−0.668 **	0.421 *	−0.427 **	0.884 **	0.221	−0.865 **	1												
bio8	0.752 **	−0.31	−0.056	−0.153	0.621 **	0.621 **	−0.308	1											
bio9	0.889 **	−0.375 *	0.276	−0.639 **	0.334 *	0.907 **	−0.751 **	0.586 **	1										
bio10	0.742 **	−0.498 **	−0.367 *	0.125	0.916 **	0.581 **	−0.113	0.760 **	0.573 **	1									
bio11	0.931 **	−0.247	0.498 **	−0.825 **	0.221	0.955 **	−0.859 **	0.575 **	0.904 **	0.456 **	1								
bio12	0.111	−0.381 *	−0.286	0.076	0.136	0.17	−0.103	−0.058	0.103	0.213	0.056	1							
bio13	0.193	−0.263	−0.067	−0.08	0.064	0.228	−0.2	0.019	0.125	0.163	0.168	0.834 **	1						
bio14	−0.14	−0.252	−0.346 *	0.226	0.042	−0.067	0.091	−0.271	−0.079	0.04	−0.175	0.833 **	0.456 **	1					
bio15	0.096	0.509 **	0.706 **	−0.501 **	−0.336 *	0.053	−0.231	0.096	0.028	−0.333*	0.258	−0.595 **	−0.149	−0.787 **	1				
bio16	0.184	−0.158	0.011	−0.099	0.055	0.191	−0.166	0.023	0.096	0.13	0.164	0.831 **	0.973 **	0.445 **	−0.1	1			
bio17	−0.128	−0.264	−0.354 *	0.224	0.051	−0.055	0.083	−0.261	−0.074	0.053	−0.166	0.836 **	0.457 **	0.999 **	−0.796 **	0.444 **	1		
bio18	−0.116	−0.032	−0.113	0.1	−0.126	−0.113	0.049	0.04	−0.175	−0.089	−0.13	0.679 **	0.725 **	0.402 *	−0.08	0.787 **	0.400 *	1	
bio19	−0.174	−0.302	−0.460 **	0.332 *	0.101	−0.111	0.167	−0.258	−0.064	0.092	−0.24	0.785 **	0.382 *	0.971 **	−0.863 **	0.359 *	0.970 **	0.321	1

Significance level: * *p* < 0.05, ** *p* < 0.01.
